# Assessing existing infection prevention and control measures used by nurses in water-scarce healthcare in a global setting: A scoping review

**DOI:** 10.4102/phcfm.v18i1.5429

**Published:** 2026-07-03

**Authors:** Makhotso M. Ralehike, Fhumulani M. Mulaudzi, Nombulelo V. Sepeng, Sinethemba Nyandeni

**Affiliations:** 1 Department of Nursing Science, Faculty of Health Sciences, University of Pretoria, Pretoria, South Africa

**Keywords:** infection prevention and control measures, hygiene practices, nurses, water scarcity, healthcare clinics

## Abstract

**Background:**

Water service in primary healthcare clinics is nearly impossible, especially in remote areas, because of poor infrastructure and ineffective municipal service delivery compliance. The health risk to patient care and safety makes basic cleanliness and infection prevention and control (IPC) practices in primary healthcare clinics difficult.

**Aim:**

This study mapped and synthesised the existing Infection Prevention and Control measures used by nurses in water-scarce healthcare facilities in a global setting.

**Method:**

A comprehensive search was conducted in four databases: EBSCOhost, Index to Nursing and Allied Health Literature (CINAHL), MEDLINE EBSCO Web of Science. Each database search employed algorithm-specific strategies and combinations. Eligible studies were those addressing IPC measures in water-scarce healthcare settings, published in English, between 2020 and 2025, and with accessible full text. Data were extracted using manual tables and thematically analysed.

**Results:**

This review identified 12 studies that met the inclusion criteria from South Asian and African countries. This review employed a descriptive qualitative methodology using thematic analysis to determine and describe the main themes that emerged from the research findings. Four themes emerged: Social behavioural strategies, good governance systems, incorporating digital and technology in healthcare systems and community participation approaches.

**Conclusion:**

This scoping review indicates that water scarcity challenges the IPC standards in healthcare facilities, adversely affecting basic hygiene practices, sanitation and overall cleanliness.

**Contribution:**

Its findings serve as a valuable resource for nurses, stakeholders, and policymakers working to improve IPC measures in primary healthcare clinics experiencing water scarcity.

## Introduction

The World Health Organization (WHO) highlights that ‘1 in 5 healthcare facilities (22%) lack basic water services, affecting 1.7 billion people, including 857 million people globally who access healthcare facilities with no water at all’.^1^ According to the WHO and UNICEF, the global report states that water is an essential public health necessity and a core component of Water, Sanitation and Hygiene (WASH) services in healthcare settings, important for infection prevention and control (IPC).^2^ The provision of safe, adequate and reliable water supply is important for all healthcare services within a primary health care (PHC) facility.^2^ Ideally, PHCs are expected to provide acute and emergency services in any circumstance. Services include obstetrics, trauma and emergency care, as well as management of both chronic and acute illnesses.^3^ Lack of water supply in PHCs disrupts health services and poses a challenge to basic health hygiene.^4^ Over 40 countries, including Saudi Arabia, Bangladesh, Bhutan and India, had adopted the WHO strategy to improve IPC in healthcare facilities facing water scarcity,^5^ the implementation of Water and Sanitation for Health Facility Improvement Tool (WASH FIT) emphasis that water services in healthcare facilities are essential for basic and life-saving IPC practices, such as hand hygiene, safe waste management and cleaning.^5^ Primary health cares are dependent on a consistent supply of clean water for operational functions, including patient care, sanitation, handwashing and the cleaning or sterilisation of medical equipment.^5,6^ In the South African Context, the Ideal Clinic Realisation and Maintenance (ICRM) framework considers the availability of clean running water as a fundamental criterion for an ‘ideal’ primary healthcare facility.^7^ However, clinics without a reliable water supply, particularly in remote areas, are prioritised for infrastructure improvements, including water storage tanks or alternative supply systems like boreholes or Jojo tanks, to ensure adherence to IPC standards and the uninterrupted operation of the clinics.^7^ However, inadequate maintenance of existing water supply systems and the failure to replace ageing infrastructures render them ineffective. Lack of water supply at PHCs disrupts services and patient care, posing challenges associated with infection in the healthcare facilities. Patients with communicable diseases are likely to be overlooked and remain untreated because of clinic closures or restricted operational hours resulting from water outrages and insufficient water supplies in the healthcare facilities. This practice has been implemented as a measure to minimise transmission of infection as a result of unhygienic conditions and poor handwash practices.^4^ The health complications of transmissible diseases can lead to epidemics and impose burdens on the healthcare system.^4^ The literature indicated several outbreaks in healthcare facilities experiencing water scarcity because of an unhygienic clinical environment and inadequate hygiene practices, which exposed both patients and nurses to healthcare-associated infections (HAIs) resulting from insufficient handwashing and general cleanliness in clinical settings.^4^ Water scarcity in healthcare facilities is an important contributor to poor-quality care and compromised basic IPC practices, as nurses are unable to adequately wash their hands, sanitise medical equipment, thoroughly clean surfaces such as examination beds or maternity tables during childbirth, and respond effectively to emergency procedures.^4,8^ Water scarcity critically impacts the health of both patients and nurses, typically posing a threat to public health. In many countries, including South Asian and African nations, water scarcity may result from extreme weather events and natural disasters such as droughts,^9^ pipe obstructions caused by chlorine in water, infrastructural degradation because of insufficient maintenance or vandalism of structures and power outages or load-shedding affecting the pumping of water from surface or underground sources.^9,10^ The failure of these systems restricts nurses from practising basic IPC practices in patient care as a result of insufficient water supply in PHCs.^10^ Water scarcity is an alarming global issue and the biggest challenge worldwide.^11^ Areas significantly impacted include India, the Middle East and countries in sub-Saharan Africa.^11^ During disease outbreaks, these countries had challenges in curbing infections and burdened health systems because of a lack of water in healthcare facilities to maintain hygiene standards.^11,12^ In reality, nurses become desperate to provide care, find themselves compelled to work in such unhygienic circumstances, resulting in them resorting to their own improvised measures, including replacing running water and handwash basins with baskets for handwashing, avoiding touching patients for physical examinations to minimise handwashing because of dry taps. Handwashing is effective in curbing infectious diseases.^13^ For instance, outbreaks of Ebola and Marburg virus in multiple countries also highlighted that health systems are underprepared and unable to respond to disease outbreaks as a result of limited access to water services and handwash facilities, a critical component of IPC during disease outbreaks.^13^ In dealing with outbreaks, WHO stated that the provision of clean water, sanitation and hygienic conditions are critical to protect human health during infectious disease outbreaks.^14,15^

Despite substantial advances in healthcare systems, 23 countries, mainly in Africa and the Eastern Mediterranean, continue to report high case fatality rates from cholera outbreaks, which are primarily because of inadequate water supply.^7,13,14,15^ Case fatality rates often exceeded the WHO-recommended threshold of < 1%, indicating systemic deficiencies in outbreak response and healthcare delivery.^14^ Contributing factors include poor IPC measures, inadequate handwashing facilities resulting from limited access to clean water.^14,15^ Efficient IPC is important for preventing HAIs and is an essential part of safe, effective, high-quality healthcare service delivery. A lack of water supply in primary healthcare clinics, particularly in remote areas, has far-reaching consequences, impacting the quality of patient care and contributing to increased death rates in various facets of nursing care.^16^ As a result, nurses are obligated to change or stop procedures in the absence of water, which directly impacts patient outcomes and safety. This means the unavailability of functional hand hygiene stations and non-adherence with hand hygiene practices because of dry taps adversely affect the health of patients. Delayed patient care, substandard sterile procedures for wound techniques and childbirth procedures will subject patients to infections resulting from unsterile practices, including inadequate hand washing and unhygienic clinical settings.^15,16^ Therefore, this review aimed to map and synthesise the existing IPC measures used by nurses in water-scarce healthcare clinics in a global setting.

## Methods

### Study design

This study applied a scoping review approach, which is a proven method for synthesising existing literature on a particular topic, emphasising key ideas or characteristics within the literature and identifying knowledge gaps.^17,18^ It employs the Arksey and O’Malley methodological framework to analyse the literature that has been relatively overlooked in existing research.^19^ The approach was revised by Levac, Colquhoun and O’Brien.^16^ This study explored the wide range of published literature on existing IPC measures used by nurses working in primary healthcare clinics facing water scarcity around the globe. A scoping review comprises five stages: Stage 1 – Formulating the research question, Stage 2 – Identifying relevant studies, Stage 3 – Selecting appropriate studies, Stage 4 – Extracting relevant data and Stage 5 – Reporting or synthesising results.^17,18^

### Research question

The research topic was: *‘What are the existing IPC measures used by nurses working in primary healthcare clinics facing water scarcity around the globe?’*

### Search strategy and eligibility criteria

This study used PCC’s framework for literature review, where P-Population comprised nurses in primary healthcare clinics; C-Content referred to IPC measures and C-Context referred to water scarcity. The search was conducted in English. The keywords used to search for articles were ‘Infection Prevention and Control measures, nurses, water-scarcity, healthcare clinics’. Four electronic databases were used for the literature search: EBSCOhost, Index to Nursing and Allied Health Literature (CINAHL), MEDLINE EBSCO and Web of Science. Each database search used different search strategies and combinations tailored for each algorithm. Filters and limitations, particularly restrictions on publication dates, articles published within 5 years period were used. A brief overview of string search is presented in Table 1.

**TABLE 1:** Search strategy based on alternative keywords.

Search string	Database	Limiters
TX (‘water-scarce’ OR ‘water scarce’ OR ‘water scarcity’ OR ‘water shortage’ OR ‘intermittent water’ OR ‘no running water’ OR ‘limited water’ OR ‘low water supply’ OR ‘restricted water’ OR ‘inadequate water’ OR ‘poor water access’ OR ‘water unavailability’) AND TX (infection control or infection prevention or infection control and prevention) AND TX (healthcare or health care or hospital or health services or health facilities) AND TX (nurses or nursing staff or nurse)	EBSCOhost Basic and/or Advanced	Open publication dates-No limitation
(infection control or infection prevention or infection control and prevention) AND (measures or interventions or steps or actions) AND (nurse or nurses or nursing) AND (water scarcity or water shortage or water crisis) AND (healthcare or health care or health services or health facilities)	MEDLINE EBSCO	Open publication dates-No limitation
infection prevention and control (All Fields) and measures (All Fields) and (nurse OR nurses OR nursing staff OR midwives OR registered nurse OR professional nurse) (All Fields) and (water scarcity OR water shortage) (All Fields) and (healthcare OR health care OR health facilities) (All Fields)	Web of Science	Open publication dates-No limitation
(infection prevention and control) AND measures AND (nurses or nursing staff or nurse) AND water scarcity AND (healthcare or health care or hospital or health services or health facilities)	CINAHL	Open publication dates-No limitation

*Source:* Ralehike MM. Assessing existing infection prevention and control measures used by nurses in water-scarce healthcare in a global setting: A scoping review. University of Pretoria; 2026.

CINAHL, Index to Nursing and Allied Health Literature.

### Inclusion and exclusion criteria

**FIGURE 1:**
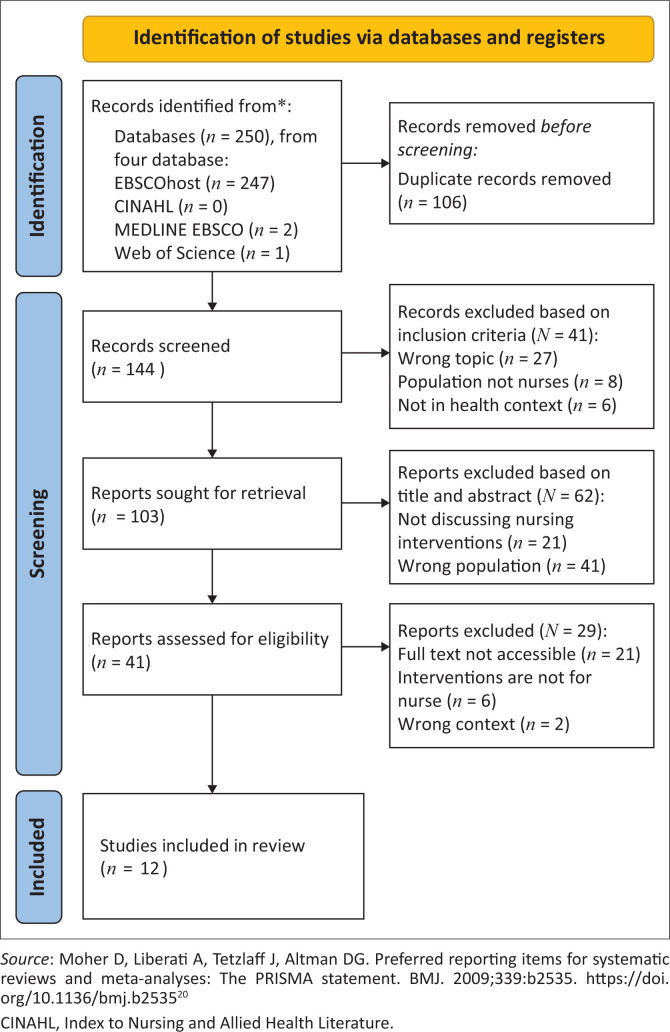
Preferred reporting items for systematic reviews and meta-analyses flow diagram.

This scoping review considered PCC (Populations, Concept, Context) to establish its inclusion and exclusion criteria, ensuring alignment with its research question. The study population included nurses in primary healthcare clinics. This study also incorporated selected peer-reviewed grey literature in the review, such as published dissertations, international organisational reports and guidelines on existing IPC measures for nurses in healthcare facilities with water scarcity. The focus ensured that the study reviewed all available literature without limiting the scope of the search or the literature itself. The search included published literature from 2020 to 2025 in the four databases. The articles were written in English, which is the language of scientific communication and writing. The aim was to assess the existing IPC measures for nurses in healthcare facilities with water scarcity across the globe. Studies were omitted if they did not relate to PCC or if the full text was inaccessible. Any publication before the year 2020 was excluded. If strategies did not address nurses in healthcare facilities experiencing water scarcity. No complete texts are accessible in English. Additional kinds of studies that were not peer reviewed include newspapers, letters, grey literature and editorials. The eligible articles were downloaded and imported into Endnote software to verify their eligibility for the screening procedure. The Preferred Reporting Items for Systematic Reviews and Meta-Analyses (PRISMA) flow diagram in Figure 1 shows the process of article inclusion and exclusion.^20^ The initial search strategy identified 250 reports obtained from online databases. Upon the removal of 106 articles, 144 articles remained. The reviewers screened 41 articles for titles that did not correspond with the research topic and were excluded. Of the 103 articles, 62 failed to meet the eligibility requirements. After an extensive review of the remaining 41 articles, a further 29 were excluded because of inadequate discussion on IPC measures for nurses in primary healthcare clinics with water scarcity. Consequently, 12 articles were incorporated into the final study. All studies were written in English. All 12 included studies explored ways for addressing water scarcity across various healthcare settings in primary healthcare clinics in different countries experiencing water scarcity.

### Data extraction

The data were manually tabulated in Microsoft Windows to show the findings of the scoping review regarding the specific characteristics of the studies. The following characteristics have been highlighted in Table 1: Author’s name, publication year, title of the study, country of research, the aim of the study, study population and findings or suggested strategies. The data screening was conducted by two reviewers to minimise bias. In the event there was no consensus, a third independent reviewer was engaged to draw a conclusion. This ensured the rigorousness of this review.

### Data analysis

This review employed a descriptive qualitative methodology using thematic analysis to determine and describe the main themes that emerged from the research findings. The two reviewers agreed on the themes that were developed. These themes were developed based on the findings of the exploration of current strategies for nurses working in primary healthcare clinics facing water scarcity. The reviewers maintained clear and transparent communication to reduce subjective biases. These measures strengthened the credibility, reliability and confirmability of the thematic analysis in this study.

### Ethical considerations

Ethical clearance to conduct this study was obtained from the University of Pretoria Faculty of Health Science Research Ethics Committee (No. 568/2025).

## Review findings

Of the twelve (*n* = 12) articles that were reviewed, see Table 2 for the details of the included articles. The studies were conducted in different countries around the globe, two in Ethopia,^21,22^ three (*n* = 3) in Nigeria,^23,24,25^ two (*n* = 2) South Asian countries,^26,27^ one (*n* = 1) India,^28^ one (*n* = 1) in different countries including Bangladesh, Burkina Faso, Chad, Guatemala, Guinea, Liberia, Malawi and Nigeria,^24^ One (*n* = 1) Pakistan,^29^ two (*n* = 2) South Africa^30,31^ and one (*n* = 1) in European countries.^32^ Four themes emerged from the scoping review: Social behavioural strategies, good governance systems, integrating digital and technology in healthcare systems and community participation approach.

**TABLE 2:** Summary of included studies.

Number	Author, year	Study title	Country	Study aim	Study population	Recommended IPC measures or strategies
1	Gebremicael et al.^21^	Implementing a multimodal intervention using local resources to improve hand hygiene compliance in a comprehensive specialised hospital in Mekelle, Northern Ethiopia	Ethiopia	This study aimed to provide insights into a strategy to implement the WHO concept adapted to local conditions and obstacles encountered at a tertiary university hospital in Mekelle, Tigray, Ethiopia.	Healthcare Workers	Implementing the WHO Hand Hygiene (HH) improvement strategy significantly increased HH compliance despite a shortage of water and other resources. Hand rub was accepted as the main HH method in the hospital. HH campaigns in developing settings benefit from multimodal strategies, knowledge exchange and utilisation of local resources.
2	Ugbah^23^	The efficacy of a community participatory process for improving access to water through the sustainability of water development projects: A case study of Okada community, Edo State, Nigeria	Nigeria	The study seeks to explore factors associated with the progress, or lack thereof, of community participation in water access in Nigeria.	Community participants located in South Nigeria	The better water access is directly linked to the high level of community participation in their water projects.
3	Berman et al.^22^	Current practices and evaluation of barriers and facilitators to surgical site infection prevention measures in Jimma, Ethiopia. Antimicrobial stewardship & healthcare epidemiology	Ethiopia	To employ the Systems Engineering Initiative for Patient Safety (SEIPS) model to better characterise surgical site infection (SSI) prevention practices and factors affecting adherence to prevention guidelines at Jimma University Medical Centre (JUMC).	Surgical nurses, surgeons and anaesthetists at JUMC	Increased training and clear guidelines will likely modestly improve adherence to Surgical Site Infection (SSI) prevention measures. Improvements in infection control behaviours are more likely to be sustained with a multimodal implementation approach that incorporates principles of behavioural theory, such as feedback, incentives and social and leadership influence. Increase IPC visibility in healthcare facilities, willing nurses can volunteer to be IPC champions tasked to regularly monitor compliance with standardised practices such as hand washing. Create friendly, interfacility competition, which has been shown to increase compliance with other infection prevention practices such as hand hygiene. Use of powder-free gloves may encourage better SSI prevention.
4	Perera et al.^26^	Reorienting health systems towards primary health care in South Asia	Five countries in South Asia: Bangladesh, India, Nepal, Pakistan and Sri Lanka.	This is the last paper in the series that outlines points for future action. We call for action in three areas. Firstly, the changing context in the region, with respect to epidemiological shifts, urbanisation and privatisation, presents an important opportunity to appraise existing policies on primary health care (PHC) and reformulate them to meet the evolving needs of communities. Secondly, reorienting health systems towards PHC requires concrete efforts on three pillars-integrated services, multi-sectoral collaboration and community empowerment.	Review of the existing literature on PHC in five South Asian countries: Bangladesh, India, Nepal, Pakistan and Sri Lanka	All five countries have taken steps to increase the availability of IPC trained health workers. All countries have community engagement processes, such as statutory health committees in decision making to strengthen the provision of public PHC services to improve comprehensiveness, integration and quality of care. All five countries have established community health worker (CHW) programmes. Overall gains in the region in the prevention and control of infectious diseases and improved maternal and child health outcomes. In some contexts, particularly in Bangladesh, NGO-led programmes have also complemented national efforts and contributed to dramatic improvements in specific areas like maternal and child health. Digital health interventions pertaining to PHC, ranging from simple to complex, are becoming increasingly prevalent in South Asia. Some examples of such interventions include applications that enable rural residents to access routine health screenings at home, telemedicine services that help connect patients to specialists, electronic patient records linked to unique health identifiers, mLearning programmes for CHWs, maternal mobile messaging programmes, mHealth audio messaging and improving health education, communication and data accuracy.
5	Kumar et al.^28^	A study on assessment of rural healthcare system in India: Schemes and implications	India	This study aims to shed light on the state of rural healthcare infrastructure, the effectiveness of government healthcare initiatives, and the far-reaching implications for rural communities.	Review of the existing literature on sub-centres (SCs), primary healthcare centres (PHCs) and community health centres (CHCs) in rural communities of India	Prioritising investments in infrastructure, healthcare personnel training and community engagement. Additionally, there is a crucial need for improved monitoring and evaluation mechanisms to ensure the efficient utilisation of resources and the impact of interventions. Collaborative efforts involving government bodies, non-governmental organisations and local communities are essential for fostering sustainable improvements in rural healthcare. Enhance community outreach, education, and preventative care. Encourage community involvement in healthcare decision-making to guarantee that offered services fulfil regional requirements. Fostering partnerships and leveraging technology. It is possible to create innovative solutions that can bridge the existing gaps and enhance the overall quality of healthcare delivery in rural India. Construct and renovate primary health centres and sub-centres, as well as other healthcare facilities, in rural areas to guarantee that essential healthcare services are accessible and available. To enable remote consultations and the sharing of medical information, improve telemedicine and the technical infrastructure. Encourage cooperation between public and private healthcare providers to raise the standard and accessibility of healthcare in rural areas. Encourage the private sector to participate in the construction and upkeep of healthcare facilities.
6	Peters et al.^24^	Resilience of front-line facilities during coronavirus disease 2019 (COVID-19): evidence from cross-sectional rapid surveys in eight low- and middle-income countries	Bangladesh, Burkina Faso, Chad, Guatemala, Guinea, Liberia, Malawi and Nigeria	This paper describes the aspects of primary health care facility resilience to COVID-19 in eight countries.	Health facility managers	Availability of handwashing stations. The infrastructure (e.g. water and sanitation) must be in place to ensure continued functioning during an emergency. Engaging with the public about public health emergencies in the healthcare facilities. Make adaptations to expand access to services (e.g. providing care in a single visit for multiple morbidities, providing home-based care, shifting clinical encounters to digital platforms and using novel dispensing approaches for medicines).
7	Khattak et al.^29^	Toward sustainable healthcare systems: A low and middle-income country’s case for investing in healthcare reforms	Pakistan	Aim to identify key strategies for achieving sustainable healthcare systems in Pakistan and to draw lessons for low- and middle-income countries (LMICs) globally, keeping in view the healthcare reforms in Pakistan.	Review of existing policies and practices related to healthcare financing, service delivery, health information and communication, governance and leadership and human resources in Pakistan and other low- and middle-income countries (LMICs)	Strengthen regulatory mechanisms, improve evidence for policy development and implementation and implement strong target-oriented monitoring and evaluation systems. Restructure all public health facilities, improve primary healthcare by incorporating behavioural health and social health determinants in the system and create highly safe and reliable healthcare organisations by incorporating national public health programmes at the district and provincial levels. Develop a policy on e-health with periodic analysis, facilitate dissemination of authentic public health information, implement electronic health records at all provincial and district health facilities, enable effective telemedicine services and develop strong ICT systems to facilitate collaboration and cooperation among health workers.
8	Hove et al.^30^	Lessons from community participation in primary health care and water resource governance in South Africa: A narrative review	South Africa	Aims to provide evidence on forms, extents, contexts and dynamics of community participation in primary health care (PHC) and water governance in South Africa and draw cross-cutting lessons.	Review on community participation in primary health care (PHC) and water governance in South Africa	Address community participation in PHC and water governance structures. Be involved in decision-making processes, identifying problems or finding solutions. Implement community-based organisations to be involved in the early planning phase, design and implementation of water delivery in PHCs.
9	Shahtaghi et al.^32^	Comparative antibacterial efficacy of formulated and commercial hand sanitisers: Formulation and evaluation	European countries	The objective of this study was to develop and assess two laboratory-developed gel-based hand sanitisers, followed by a clinical trial.	Healthcare workers	In both healthcare and community settings, hand sanitisers and/or hand rubs, especially antimicrobial hand rubs, have gained popularity as a viable substitute for traditional handwashing with soap and water by claiming to be effective against harmful microbes. Alcohol-based hand sanitisers are recommended for hand hygiene in clinical settings to prevent infections.
10	Luby et al.^27^	Broad approaches to cholera control in Asia: Water, sanitation and handwashing	Asia	The objective of this paper is to reflect on the importance of complementary strategies to reduce the burden of cholera and other infectious water-borne diseases, and to consider how such efforts can be simultaneously advanced to work towards decreasing the burden of disease.	Low-income communities at the highest risk for cholera	Hospitals and clinics should have water supplies that are not contaminated by human faeces, as well as easily accessible locations to wash hands with soap. Using soapy water made from low-cost detergent or developing other inexpensive approaches can help address these barriers. Approaches other than soap and water may extend the reach of hand hygiene, especially in water-scarce contexts, but these approaches need to be remarkably low cost to reach people at the highest risk. Well-functioning centralised water distribution systems require capital for construction and funds for ongoing maintenance and repair, municipal water suppliers in weak states commonly need the incentives and capacity to regularly collect payments.
11	Uchenna et al.^25^	Scarcity of quality water supply as a facility management challenge in primary health care centres in Nigeria: A case study of Anambra State	Nigeria	The study aimed to assess the extent of quality water scarcity in PHCs in Anambra State, identify its causes, evaluate its effects on the facility management and propose improvement strategies.	PHC staff members	The government should increase budgetary allocations for water infrastructure, healthcare facilities should adopt solar-powered boreholes, facility managers should implement regular maintenance schedules and public-–private partnerships with community involvement should be promoted to ensure a sustainable water supply. One key strategy involves the implementation of sustainable water infrastructure, including boreholes and rainwater harvesting systems, which can mitigate dependence on erratic municipal supplies. Interventions must be complemented by routine maintenance protocols to prevent system failures, a factor often overlooked in low-resource contexts. PHCs should adopt point-of-use water treatment technologies, such as chlorination or filtration systems, to address immediate contamination risks. These measures, however, require consistent funding and technical capacity. Involving local stakeholders in monitoring and reporting water supply issues can enhance responsiveness. Strengthening intersectoral collaboration between health, water and environmental agencies. Public–private partnerships to mobilise resources for PHC water projects, particularly in urbanising areas. Diversifying water sources, such as incorporating solar-powered desalination units, to mitigate climate risks. Incorporating technology for real-time water supply monitoring can enhance efficiency and accountability. Sensor-based systems to detect leaks or contamination promptly, reducing downtime and health risks. Community participation remains a cornerstone of sustainable water supply strategies. PHCs must collaborate with local leaders and civil society groups to foster collective ownership of water projects. Community-based water committees can play a pivotal role in maintaining infrastructure and enforcing usage guidelines.
12	Matimolane et al.^31^	Tackling rural water scarcity in South Africa: climate change, governance and sustainability pathways	South Africa	Synthesises secondary data through the Integrated Water Resources Management (IWRM) and socio-ecological systems (SES) frameworks, offering a novel lens that bridges macro-policy failures with micro-community resilience, unlike single-framework analyses.	Existing case studies	Adopting emerging technologies such as AI-enabled monitoring. Implementation of rainwater harvesting systems, by collecting and storing rainwater, can help households and schools to reduce their reliance on municipal water systems. The installation of rainwater harvesting tanks has proven to be a cost-effective and sustainable solution for many rural communities. Solar-powered water purification technologies are providing a reliable and sustainable solution for rural communities with limited access to electricity. Use of solar-powered reverse osmosis systems in remote areas, which helps purify contaminated water and provide clean drinking water to off-grid communities. In South Africa, several community-driven initiatives have been launched to encourage local participation in water conservation. Programmes like Water Wise, led by the WRC, educate communities on how to reduce water consumption and adopt water-saving practices. The campaign has reached millions of South Africans, encouraging practices such as fixing leaking taps, using water-efficient appliances and promoting rainwater harvesting.

Note: Please see the full reference list of the article, Ralehike MM, Mulaudzi FM, Sepeng NV, Nyandeni S. Assessing existing infection prevention and control measures used by nurses in water-scarce healthcare in a global setting: A scoping review. Afr J Prm Health Care Fam Med. 2026;18(1), a5429. https://doi.org/10.4102/phcfm.v18i1.5429, for more information.

WHO, World Health Organization; ICT, Information and Communication Technology; WRC, Water Research Commission.

### Theme 1: Social behavioural strategies

#### Sub-theme 1.1: Hand hygiene practices

Hand hygiene is considered the most effective IPC measure and is endorsed by several countries as a critical standard of care. The first article, conducted in Asia, indicated that the implementation of hygiene practices, particularly emphasising the need for hand hygiene for nurses, may be enhanced by providing resources such as soap and running water.^32^ In the absence of water availability, the study conducted by Luby and colleagues suggested that the use of soapy water made from low-cost detergents or any other inexpensive approaches should be used in Asia for basic hand hygiene.^27^ In contrast, several studies found that regular use of handrub steriliser is utilised in European nations and Ethiopia to maintain hand hygiene standards.^21,22,32^ Countries, including Liberia and Malawi, emphasised the significance of handwashing stations in the healthcare facilities to ensure good hygiene.^24^

#### Sub-theme 1.2: Hygiene champions

Hygiene champion is a strategy to encourage the compliance of hygiene practices, such as handwashing and cleaning quality, to improve prevention of infection and raise safety standards. Nurses nominate one of their colleagues in healthcare facilities to act as a hygiene champion to serve as a leader and trainer to oversee the hygiene compliance. In support of these findings, the study conducted by Berman and colleagues suggested that in Ethiopia, nurses volunteer to be IPC champions to improve handwashing and general cleanliness compliance.

#### Sub-theme 1.3: Knowledge empowerment

Several studies conducted in nations including Ethiopia, Guatemala and Guinea emphasised the importance of training nurses, patients and the community on hygiene as a measure to improve IPC in healthcare facilities experiencing water scarcity.^21,22^ In support of these findings, the study conducted by Gebremicael et al., in Ethiopia, indicated that knowledge exchange has been beneficial for hand hygiene compliance in healthcare facilities that have a water shortage.^21^

### Theme 2: Good governance systems

#### Sub-theme 2.1: Water sources and systems

Uninterrupted water supply is suggested in a number of studies as significant to ensure IPC is improved in healthcare facilities. The study conducted in India by Kumar and others suggested that the maintenance of infrastructures.^28^ Similarly, in Nigeria, the study emphasised the implementation of water infrastructure, including boreholes and rainwater harvesting systems, to mitigate IPC standards in healthcare facilities.^25^

Additionally, the study conducted in Asia suggested the availability of funds to support municipalities for the maintenance and repair of water suppliers to ensure water is available in the facilities.^36^ In support of this finding, the study conducted in South Africa recommends the fixation of leaking taps and the use of water-efficient appliances to promote water supply.^31^

### Theme 3: Integrating digital and technology in the health system

The majority of countries incorporated technology into healthcare systems for evaluation and monitoring purposes. In certain regions of South Africa, including the Cape Town province, given that electricity is a challenge in South Africa, the utilisation of solar power serves as a contingency for water delivery, ensuring that PHCs continue to have a supply of water.^31^ Similar to that, countries such as Pakistan, Nigeria and Bangladesh have implemented telemedicine systems and digital platforms like e-health and mHealth audio messaging to enhance healthcare services and minimise clinic visits.^26,24,29,25^ In several countries, including Burkina Faso, Chad, Guatemala, Guinea, Liberia and Malawi, patients obtain multiple services within a single clinic visit, utilise innovative medication dispensing systems for stable patients, thereby implementing methods to enhance access to health services in facilities experiencing water scarcity.^24^

Furthermore, the study conducted in South Africa suggested the implementation of technology such as Artificial Intelligence (AI) devices to monitor water supply in healthcare facilities, as a measure to ensure that water taps are programmed to dispense clean water during handwashing.^31^

### Theme 4: Community participation approach

Several nations in South Asia and South Africa emphasised the importance of community engagement and collaboration with external sub-sectoral stakeholders in water access strategies to address compromised IPC measures in healthcare facilities experiencing water scarcity.^26,30,31^ Five South Asian countries, including Bangladesh, India, Nepal, Pakistan and Sri Lanka, have redirected healthcare services to the community by implementing outreach programmes.^26^ These initiatives include home-based care visits, mobile clinics for remote regions and the implementation of Community Healthcare Workers (CHW) programmes for routine screenings and follow-ups, aimed at alleviating the burden on the healthcare system during water shortages.^26^ This is a key measure for controlling infections acquired in medical facilities during periods of water constraint. In particular contexts, notably in Bangladesh and India, non-governmental organisations (NGOs) are supporting programmes related to maternal and child health in specific regions.^26,28^ In Nigeria, it has been suggested that community health committees, as part of the PHC team, would serve as a representative voice for both nurses and the community in enforcing IPC standards in healthcare facilities experiencing water scarcity.^25^ The health committee will engage communities in decision-making, encourage their participation in PHC and water governance structures to strengthen the provision of public PHC services and improve quality care.^25^ These studies collectively highlighted existing IPC practices that nurses in healthcare facilities experiencing water scarcity globally might adopt to provide quality patient care to ensure the continuation of care.

### Discussion of findings

This section presents a discussion of the main findings from the literature review, arranged into four themes: social behavioural strategies, good governance systems, incorporating digital and technology in healthcare systems and community participation approaches.

#### Theme 1: Social behavioural strategies

The results of this review revealed that adopting social behavioural strategies such as consistent hand hygiene practices, selecting hygiene champions and knowledge empowerment of nurses would ensure safe practices and prevent infection in healthcare clinics facing water scarcity. This finding supports nurse’s innovative approaches to maintain safe practices despite the limited availability of water. In Germany, these automated dispensers had shown a reduction in contamination and an improvement in hand hygiene in water-scarce healthcare facilities.^33^ Similarly, in the United States, sensor-based water taps have been introduced to minimise water flow and waste. These smart water management systems have facilitated better leakage detection and contribute to water conservation. In addition, the use of alcohol-based hand rubs (ABHR) should be prioritised as the primary method for hand hygiene when hands are not visibly soiled.^33,34^ as it is effective in killing microbes that cause infection. The installation of these touch-free ABHR dispensers should be strategically placed in points of care, including bedside, procedure rooms, consulting rooms and reception or entrance areas, to improve compliance. Ensuring that nurses are adequately skilled and trained in innovative practices would promote effective IPC measures in primary healthcare facilities.

#### Theme 2: Good governance systems

**FIGURE 2:**
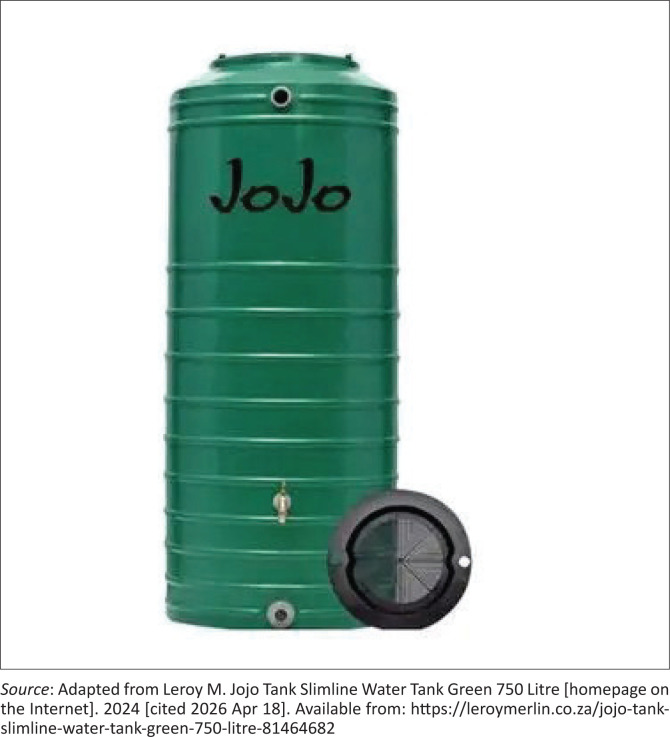
Jojo tank connected with a tap.^35^

The findings of this review indicated that water supply must be readily available and accessible in healthcare facilities, particularly in remote PHCs, as they are often the sole healthcare providers in the areas they serve.^4^ In support of these findings, to ensure improved IPC measures, water supply to healthcare facilities should be facilitated in communities through reliable funding to pay water suppliers and empowering a well-coordinated and effective local authority to manage water services.^25,28^ This study was supported by studies conducted in many countries experiencing water scarcity, including Nigeria, South Africa and Nepal, where the nurses in healthcare facilities depend on water supply sources such as boreholes, communal tanks yard taps, rainwater harvesting systems (JoJo tanks) (Figure 2) and water tankers during water shortages to sustain operations; these adaptive measures should be preserved.^25,27,31^ Ensuring clean running water in healthcare is a public good,^1,2,3^ and in the event of a water outage, the local municipality should intervene to supply water to PHCs.^27^

Furthermore, even though nurses rely on the availability of water to ensure good hand hygiene and general cleanliness of the clinic, the feasibility of access to water relies on municipalities getting professional skills in managing the finances of water systems by generating revenue. This might assist in facilitating regular maintenance, infrastructural advancements and productivity improvements in clinics, which will guarantee a continuous water supply for quality patient care.^24,28,30^ Based on the findings in this review, the researcher believes that in some regions where water shortage is both irreversible and progressive, municipalities must consistently refill communal tanks, particularly at healthcare facilities in remote areas. No clinic or PHC should be closed as a result of poor governance of its water services. On the other hand, this review is in support of the importance of maintaining water-related infrastructure to ensure consistent access to clean running water in healthcare facilities. Similarly, the study conducted by Sharma and colleagues and Iversen and others states that regular maintenance and checking of water storage containers, repair of leaking taps and timely restoration of water supply pipelines and systems will all ensure a reliable water supply.^36,37^ In support of this claim, this review discovered that upgrading ageing infrastructures and installing backup water sources in healthcare facilities will protect emergency water storage from contamination. These measures will ensure access to clean water and ensure that IPC standards are maintained in healthcare facilities.

#### Theme 3: Integrating digital and technology in the health system

**FIGURE 3:**
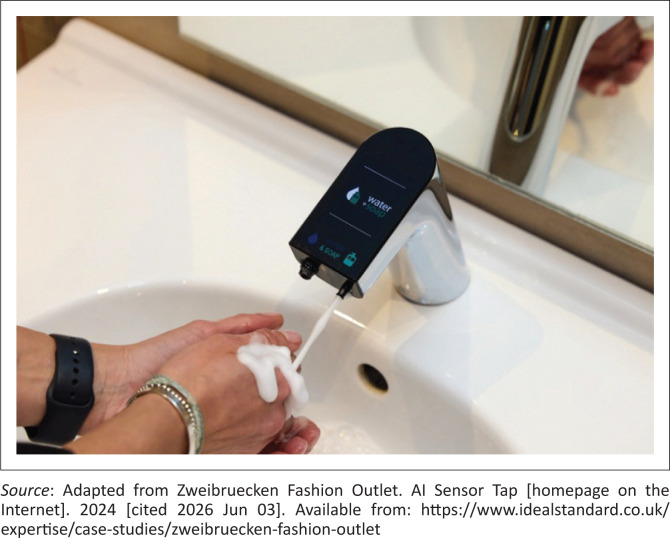
Artificial intelligence sensor tap.^43^

Integrating technology into healthcare is important, particularly in this era of water scarcity. The implementation of tippy taps (Figure 3), which are low-flow taps, regulates water flow to minimise waste.^38^ This strategy is adopted in several countries to improve handwashing. Tippy taps were approved by UNICEF and WaterAid in Uganda and are extensively used in India, Mozambique, Tanzania and Zambia.^38^ The implementation of this handwashing approach enhances hand hygiene in regions experiencing water constraints. A study conducted in African nations, including Mozambique and Zambia, indicates that this locally made water-saving handwashing device is easy to use and cost-effective.^38^ The introduction of other devices, including automated or manually controlled foot-operated or elbow-operated taps, will help minimise water waste. Although automated is a costly alternative that may be unfeasible for many facilities because of its electricity demands and complex design.^39^ Additionally, Pakistan uses a manual foot-elbow-operated handwashing system, which is more feasible for the majority of healthcare facilities in the country.^39^ In contrast, these measures are mostly influenced by cultural practices, technologies and advancements. For instance, most Western countries, including the United States, Europe and Australia, have introduced water-conserving sanitation technologies, such as bio-digesters or low-flush toilets, commonly referred to as ‘e-toilets’, which require less maintenance than traditional models.^40^ These measures are meant to promote effective IPC practices in healthcare facilities experiencing water scarcity in developed countries. In comparison to some Asian countries, traditional toilet practices frequently employ manual cleaning methods, such as the use of buckets, as a means to conserve water in both rural and urban areas.^39,40^ Healthcare clinics need a continuous and uninterrupted energy supply to maintain the operation of water sources, such as pressure pumps, important for delivering health services.^36^ The switch to renewable energy sources, such as solar, thermal and hydroelectric energy, in developing nations like China, Europe, Japan and the United States, seems to be the most feasible approach to ensure standardised IPC in healthcare facilities facing water scarcity.^28,41^ Water supply in PHCs is important in achieving global environmental and WASH sustainability. The introduction of solar-powered portable handwashing stations in Nigeria provides more effective hand hygiene solutions for healthcare facilities that experience water scarcity.^42^ This system allows touchless handwashing in public areas, utilising sensors to automatically dispense soap, water and even allow hand drying (Figure 3).^42^ This approach would ensure proper hand hygiene in PHCs experiencing water scarcity while also reducing overall water consumption.

Minimising the number of patients physically attending the PHCs reduces water consumption. In support of this, South Asian nations, particularly Pakistan, have implemented telemedicine and digital routine screening systems.^44^ This remote technology facilitates follow-up care and counselling.^44^ Similarly, in developed countries, including the United States, patients receive their monthly chronic medication from retailers affiliated with local health departments.^44^ These initiatives, supported by active community engagement, assist in minimising patient exposure to HAIs resulting from insufficient IPC practices because of a lack of water at the facilities.

#### Theme 4: Community participation approach

**FIGURE 4:**
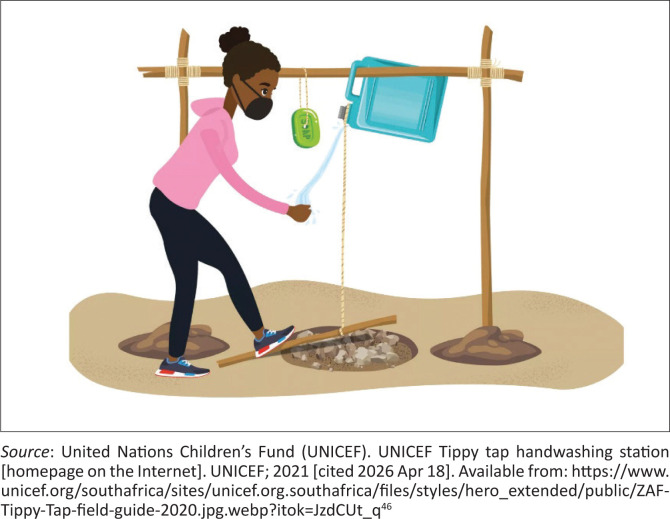
UNICEF’s Tippy Tap handwash demonstration guide.

**FIGURE 5:**
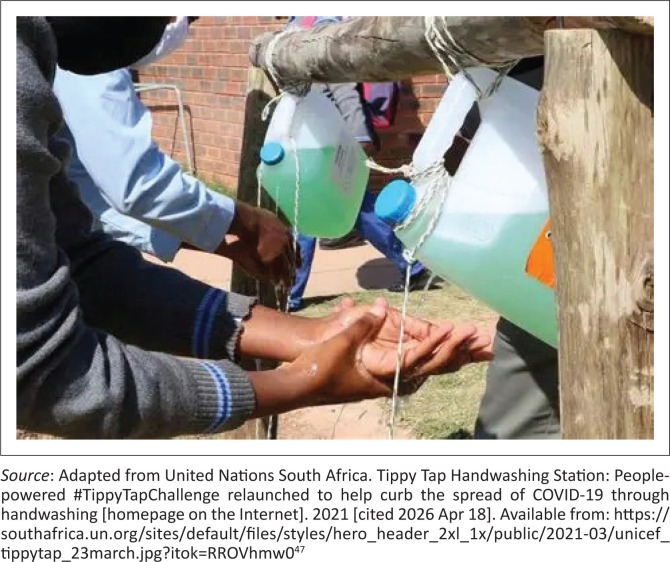
Tippy taps handwash station.^47^

Community involvement in water projects is essential.^20,26,28^ Number of studies support that community stakeholders must collaborate with planners at all levels of water project planning.^29,41^ In this review, the researcher is of the view that involving nurses in strategic development is essential; this joint decision will ensure their commitment. Furthermore, this participatory decision-making demonstrated that nurses would appreciate these strategies and show support for implementing the interventions to ensure standardised IPC practises are in place.^26,28^ The findings indicate that the conditions might improve throughout the preparation and implementation of these IPC measures in healthcare facilities, if implementing authorities engage communities in the provision of water services, allowing community members, particularly nurses, to express their ideas on their preferred water strategies.^23,29,41^ Hence, certain water sources may be ineffective for some facilities while being beneficial for others. Nurses and other community members should be engaged in all levels of decision-making to promote participation and community governance.^26,28,24^ Literature indicates that the inclusion of end-user participation is essential during the planning and development of water access projects.^23^ Consequently, they will be able to advise on the most effective strategies to be applied in their community, particularly with water strategies to address IPC. The collaborative sharing of ideas and knowledge strengthens the relationship between water management providers and the end user, namely nurses in PHCs.^23^ Additionally, different government departments should collaborate with other sectors to ensure primary healthcare clinics have access to a reliable and sustainable water supply for patient care and IPC practices.^26,28,29^ Primary healthcare in numerous lower- and middle-income nations, including those in Sub-Saharan Africa, such as Ethiopia, Uganda and Niger, as well as Asian countries like Nepal and Madagascar, relies on critical interventions from water aid organisations from the World Bank for water supply. Several studies indicate that community engagement promotes accountability and sustainability in addressing water scarcity and IPC in healthcare facilities.^21,22,28,24^ Community awareness and education can significantly contribute to water conservation practices.^25^ Collaboration with local municipalities, NGOs and community leaders to support rainwater harvesting around the health facilities, as well as the installation of communal water stations, preferably located close to the healthcare facility, will help strengthen community-driven solutions to water scarcity.^25^ Building on this collaborative approach, community volunteer initiatives to support PHCs are implemented in countries such as Nigeria, where trained volunteers provide basic health services within communities, including home visits for palliative and chronically ill patients.^25,45^ This approach helps reduce unnecessary clinic attendance for routine follow-ups, thereby lowering the strain on limited water resources. In addition, this review suggests that communities can also be encouraged to create low-cost hand hygiene solutions, such as tippy taps (Figure 4 and Figure 5) or handwashing buckets, to support the PHCs in maintaining proper hand hygiene during periods of water scarcity.

Water scarcity presents a significant IPC challenge for healthcare facilities, particularly PHCs in many countries, including those in Asia, Nepal, India and South Africa. Nurses, in collaboration with communities and other stakeholders, can help minimise health risks through adaptable hand hygiene practices, ensuring proper maintenance of infrastructure, integrating suitable technologies and engaging community support.

## Implications and recommendations

This study supports the findings of the reviewed literature, indicating the vital role of community engagement in strengthening IPC measures for nurses working in PHCs experiencing water scarcity around the globe. The introduction of water-saving devices can improve hand hygiene in healthcare facilities with water constraints globally. These solutions include tippy taps, handwashing buckets, alcohol-based hand rub dispensers, automated and manual foot- and elbow-operated handwashing systems, along with various other behavioural strategies to address hygiene in PHCs facing water scarcity. Incorporating suitable technology can further support hand hygiene, sanitation and general cleanliness of the clinics, helping maintain safe and standardised IPC practices during periods of water scarcity. This review strongly recommends the development of strategies to guide infection prevention and control policy for nurses in water-scarce healthcare facilities

## Limitations

This study reviewed articles from various countries globally, highlighting a cross-knowledge gap on this topic for the period of 5 years. The limitation of the study is that its findings were focused on primary healthcare settings; therefore, its findings cannot be generalised to a broader population. Although the conclusions are not generalised, they can be used in other contexts to improve the implementation of IPC during periods of water scarcity.

## Conclusion

Infection prevention and control standards are compromised when healthcare facilities are experiencing water scarcity, impacting the quality-of-care nurses render. Incorporating behavioural strategies such as health education might improve knowledge on hygiene practices in settings where there is water scarcity for both nurses and patients. The support of healthcare technologies in nursing will enhance patient care and improve hygiene practices in regions where water scarcity is a challenge. This review highlights the significance of participatory action with nurses and the community when addressing issues such as IPC in the context of water scarcity.

## References

[1] World Health Organization (WHO) & UNICEF. Progress on WASH in health care facilities 2000–2021: Special focus on WASH and infection prevention and control (IPC) [homepage on the Internet]. 2023 [cited 2025 Sep 16]. Available from: https://apps.who.int/iris/handle/10665/366657

[2] World Health Organization (WHO). Drinking-Water [homepage on the Internet]. 2023 [cited 2025 Sep 16]. Available from: https://www.who.int/news-room/fact-sheets/detail/drinking-water

[3] World Health Organization. Progress on WASH in health care facilities 2000–2021: Special focus on WASH and infection prevention and control (Global report) [homepage on the Internet]. 2023 [cited 2025 Sep 18]. Available from: https://www.who.int/publications/i/item/9789240058699

[4] Berihun G, Adane M, Walle Z, et al. Access to and challenges in water, sanitation, and hygiene in healthcare facilities during the early phase of the COVID-19 pandemic in Ethiopia: A mixed-methods evaluation. PLoS one. 2022;17(5):e0268272. 10.1371/journal.pone.0268272 35560168 PMC9106162

[5] World Health Organization (WHO). Water and sanitation for health facility improvement tool (WASH FIT): A practical guide for improving quality of care through water, sanitation and hygiene in health care facilities [homepage on the Internet]. 2nd ed. 2022 [cited 2025 Sep 18]. Available from: https://apps.who.int/iris/handle/10665/353411

[6] World Health Organization (WHO) & UNICEF. Water, sanitation, hygiene, waste and electricity services in health care facilities: Progress on the fundamentals [homepage on the Internet]. 2023 [cited 2025 Sep 18]. Available from: https://iris.who.int/server/api/core/bitstreams/f11714c7-65ec-4328-a454-cda91ba0a084/content

[7] The Ideal Clinic Realisation and Maintenance (ICRM) Framework. Ideal clinic and community health centre Tm definitions, components and checklists [homepage on the Internet]. 2025 [cited 2025 Oct 01]. Available from: https://www.bing.com/search?q=Ideal%20Clinic%20Realisation%20and%20Maintenance%20(ICRM)%20framework&form=SWAUA2

[8] WaterAid Uganda country programme strategy 2023–2028 [homepage on the Internet]. 2023 [cited 2026 May 19]. Available from: https://www.wateraid.org/washmatters/our-work/health

[9] Mahato A, Upadhyay S, Sharma D. Global water scarcity due to climate change and its conservation strategies with special reference to India: A review. Plant Arch. 2022;22(1):64–69. 10.51470/PLANTARCHIVES.2022.v22.no1.009

[10] Jiyane MP, Khunou SH. Experiences of professional nurses regarding shortage of resources at a tertiary hospital in Gauteng Province, South Africa: Qualitative study. Jurnal Ners. 2023;18(2):176–183. 10.20473/jn.v18i2.44792

[11] WHO and UNICEF. WASH in health care facilities 2023 data update: Special focus on primary health care. WHO/UNICEF Joint Monitoring Programme (JMP) for Water Supply, Sanitation and Hygiene (WASH) [homepage on the Internet]. 2024. Available from: https://data.unicef.org/resources/jmp-wash-in-health-care-facilities-2024/

[12] Adom R. Analysis of public policies and programmes to water security in post-apartheid South Africa. Johannesburg: University of the Witwatersrand; 2020.

[13] World Health Organization (WHO). Cholera: Global situation [homepage on the Internet]. 2023 [cited 2025 Oct 01]. Available from: https://www.who.int/emergencies/disease-outbreak-news/item/2023-DON437

[14] Ogunniyi TJ, Muoneke AP, Nimo F, Yisa SS, Olorunfemi OA. Cholera in Nigeria: A five-decade review of outbreak dynamics and health system responses. J Health Popul Nutr. 2025;44(1):329. 10.1186/s41043-025-01096-7 41024210 PMC12482021

[15] Challa JM, Getachew T, Debella A, et al. Inadequate hand washing, lack of clean drinking water and latrines as major determinants of cholera outbreak in Somali region, Ethiopia in 2019. Front Public Health. 2022;10:845057. 10.3389/fpubh.2022.845057 35602140 PMC9120658

[16] Malematja DN, Nkosi EM, Nene SE. The impact of insufficient resources on the quality-of-service delivery at a primary healthcare clinic in Limpopo. Curationis. 2025;48(1):1–7. 10.4102/curationis.v48i1.2696 PMC12067016 40336378

[17] Bitomsky L, Pfitzer EC, Nißen M, Kowatsch T. Advancing health equity and the role of digital health technologies: A scoping review protocol. BMJ Open. 2024;14(10):e082336. 10.1136/bmjopen-2023-082336 PMC11481109 39414274

[18] Yosep I, Mardhiyah A, Hazmi H. et al. A scoping review of nursing interventions for reducing the negative impacts of domestic violence among women. BMC Nurs. 2024;23:834. 10.1186/s12912-024-02453-3 39543631 PMC11566146

[19] Arksey H, O’malley L. Scoping studies: Towards a methodological framework. Int J Soc Res Methodol. 2005;8(1):19–32. 10.1080/1364557032000119616

[20] Moher D, Liberati A, Tetzlaff J, Altman DG. Preferred reporting items for systematic reviews and meta-analyses: The PRISMA statement. BMJ. 2009;339:b2535. 10.1136/bmj.b2535 19622551 PMC2714657

[21] Gebremicael MN, Skaletz-Rorowski A, Potthoff A, et al. Implementing a multimodal intervention using local resources to improve hand hygiene compliance in a comprehensive specialized hospital in Mekelle, Northern Ethiopia. Int J Hyg Environ Health. 2024;259:114389. 10.1016/j.ijheh.2024.114389 38703463

[22] Berman LR, Lang A, Gelana B, et al. Current practices and evaluation of barriers and facilitators to surgical site infection prevention measures in Jimma, Ethiopia. Antimicrob Steward Healthc Epidemiol. 2021;1(1):e51. 10.1017/ash.2021.227 36168452 PMC9495540

[23] Ugbah RN. The efficacy of a community participatory process for improving access to water through the sustainability of water development projects: A case study of Okada community, Edo State Nigeria [Doctoral dissertation]. Glasgow Caledonian University; 2022.

[24] Peters MA, Ahmed T, Azais V, et al. Resilience of front-line facilities during COVID-19: Evidence from cross-sectional rapid surveys in eight low-and middle-income countries. Health Policy Plan. 2023;38(7):789–798. 10.1093/heapol/czad032 37256762 PMC11318646

[25] Uchenna AO, Elohor A. Scarcity of quality water supply as a facility management challenge in primary health care centres in Nigeria: A case study of Anambra State. IRE J. 2025;8(12):1594–1608.

[26] Perera S, Ramani S, Joarder T, et al. Reorienting health systems towards primary health care in South Asia. Lancet Reg Health-Southeast Asia. 2024;28:100466. 10.1016/j.lansea.2024.100466 39301269 PMC11410733

[27] Luby SP, Davis J, Brown RR, Gorelick SM, Wong TH. Broad approaches to cholera control in Asia: Water, sanitation and handwashing. Vaccine. 2020;38:A110–A117. 10.1016/j.vaccine.2019.07.084 31383486

[28] Kumar JS, Shobana D. A study on assessment of rural health care system in India: Schemes and implications. Economics. 2024;12(2):24–34. 10.34293/economics.v12i2.7166

[29] Khattak AF, Rahman AU, Khattak M, et al. Toward sustainable healthcare systems: A low and middle-income Country’s case for investing in healthcare reforms. Cureus. 2023;15(5):e39345. 10.7759/cureus.39345 37351239 PMC10284437

[30] Hove J, D’Ambruoso L, Kahn K, et al. Lessons from community participation in primary health care and water resource governance in South Africa: A narrative review. Glob Health Action. 2022;15(1):2004730. 10.1080/16549716.2021.2004730 34994680 PMC8745361

[31] Matimolane S, Mathivha FI. Tackling rural water scarcity in South Africa: Climate change, governance, and sustainability pathways. Front Environ Sci. 2025;13:1550738. 10.3389/fenvs.2025.1550738

[32] Shahtaghi NR, Bigdelitabar S, Thakur S, et al. Comparative antibacterial efficacy of formulated and commercial hand sanitizers: Formulation and evaluation. J Adv Zool. 2023;44(S3):839–850. 10.17762/jaz.v44iS-3.812

[33] Reed M, Huang J, Graetz I, Muelly E, Millman A, Lee C. Treatment and follow-up care associated with patient-scheduled primary care telemedicine and in-person visits in a large integrated health system. JAMA Netw Open. 2021;4(11):e2132793. 10.1001/jamanetworkopen.2021.32793 34783828 PMC8596201

[34] Dick A, Sterr CM, Dapper L, Nonnenmacher-Winter C, Günther F. Tailored positioning and number of hand rub dispensers: The fundamentals for optimized hand hygiene compliance. J Hosp Infect. 2023;141:71–79. 10.1016/j.jhin.2023.08.017 37660889

[35] Leroy M. Jojo Tank Slimline Water Tank Green 750 Litre [homepage on the Internet]. 2024 [cited 2026 Apr 18]. Available from: https://leroymerlin.co.za/jojo-tank-slimline-water-tank-green-750-litre-81464682

[36] Mulopo C, Chimbari MJ. Water, sanitation, and hygiene for schistosomiasis prevention: A qualitative analysis of experiences of stakeholders in rural KwaZulu-Natal. J Water Sanit Hyg Dev. 2021;11(2):255–270. 10.2166/washdev.2021.182

[37] Iversen AM, Hansen MB, Münster M, Kristensen B, Ellermann-Eriksen S. Hand hygiene compliance in nursing home wards: The effect of increased accessibility of alcohol-based hand rub. J Hosp Infect. 2024;147:206–212. 10.1016/j.jhin.2024.02.027 38521416

[38] Olatunde TM, Adelani FA, Sikhakhane ZQ. A review of smart water management systems from Africa and the United States. Eng Sci Technol J. 2024;5(4):1231–1242. 10.51594/estj.v5i4.1014

[39] Mbakaya BC, Kalembo FW, Zgambo M. Use, adoption, and effectiveness of tippy-tap handwashing station in promoting hand hygiene practices in resource-limited settings: A systematic review. BMCPublic Health. 2020;20(1):1005. 10.1186/s12889-020-09101-w PMC7316639 32586314

[40] Sarwar S, Muhammad J, Shahzad F. A modified hand-washing method for resource limited settings. Front Public Health. 2022;10:965853. 10.3389/fpubh.2022.965853 35991070 PMC9386356

[41] Sharma L, Singh J, Dhiman R, et al. Advancing solar energy for primary healthcare in developing nations: Addressing current challenges and enabling progress through UNICEF and collaborative partnerships. Cureus. 2024;16(1):e51571. 10.7759/cureus.51571 38313940 PMC10835743

[42] Narone O. Sewage treatment of self sustainable e-toilets using bio-digester method. Int J Res Appl Sci Eng Technol. 2022;10(6):4408–4414. 10.22214/ijraset.2022.44904

[43] Zweibruecken Fashion Outlet [homepage on the Internet]. AI Sensor Tap. 2024 [cited 2026 Jun 03]. Available from: https://www.idealstandard.co.uk/expertise/case-studies/zweibruecken-fashion-outlet

[44] Animashaun LA, Yekinni AA, Adigun MA, Akhiwolo C, Osinoiki AA. Development and prototyping of sensor-based touchless handwashing and drying system. Laspotech J Sci Eng Technol Res. 2023;2(1):41–53.

[45] Lin JC, Kavousi Y, Sullivan B, Stevens C. Analysis of outpatient telemedicine reimbursement in an integrated healthcare system. Ann Vasc Surg. 2020;65:100–106. 10.1016/j.avsg.2019.10.069 31678131

[46] United Nations Children’s Fund (UNICEF). Tippy tap handwashing station [homepage on the Internet]. UNICEF; 2021 [cited 2026 Apr 18]. Available from: https://www.unicef.org/southafrica/sites/unicef.org.southafrica/files/styles/hero_extended/public/ZAF-Tippy-Tap-field-guide-2020.jpg.webp?itok=JzdCUt_q

[47] United Nations South Africa. Tippy Tap Handwashing Station: People-powered #TippyTapChallenge relaunched to help curb the spread of COVID-19 through handwashing [homepage on the Internet]. 2021 [cited 2026 Apr 18]. Available from: https://southafrica.un.org/sites/default/files/styles/hero_header_2xl_1x/public/2021-03/unicef_tippytap_23march.jpg?itok=RROVhmw0

